# Access to Inclusion Thinking Beyond Reasonable Adjustments

**DOI:** 10.1111/hex.70157

**Published:** 2025-01-16

**Authors:** Sandra Paget, Agata Pacho

**Affiliations:** ^1^ PPI Contributor UK; ^2^ Department of Health Services Research & Policy London School of Hygiene and Tropical Medicine London UK

## Introduction

Disabled public contributors continue to experience challenges with inclusion, despite inclusive patient and public involvement (PPI) being regarded as the gold standard in research practice and despite disabled PPI contributors often making up a significant proportion of all public contributors [1]. With the recent Disability Framework from the National Institute for Health and Care Research (NIHR), which commits to disability inclusion in health and healthcare research [1], it is timely to reflect on the current situation.

This article is written in the first person by Sandra Paget and Agata Pacho, who share their perspectives as a PPI contributor and an academic leading PPI, respectively. Sandra is an experienced reviewer for the NIHR and has collaborated with academics across the UK. Having lived with a rare neurological condition since early childhood, she founded the original Buckinghamshire Neurological Alliance and served on the Executive Committee of the National Neurological Alliance, representing Regional Neurological Alliances. In this article, Sandra reflects on instances where her ability and willingness to contribute to research were limited by accessibility barriers or a lack of adequate access. She argues that these barriers not only affect individual PPI activities but also have a long‐term impact on healthcare services, making it harder for disabled people to live independently for longer. Agata, an Assistant Professor at the London School of Hygiene & Tropical Medicine (LSHTM), leads PPI for the NIHR Policy Research Unit in Policy Innovation and Evaluation (PIRU). Here, Agata discusses how the notion of reasonable adjustments may, in certain cases, be unhelpful or even counterproductive to fostering inclusivity within PPI. Instead, Agata suggests that embedding a duty of care into how PPI is conducted could be a more effective way to break down barriers and ensure greater accessibility for disabled contributors.

### Sandra: No Access to Inclusion

I am a part of the infamous movement of public contributors to the research—a movement that is facing the crucial challenge of ensuring inclusion. Whenever I am invited anywhere the first question that comes to my mind is, ‘can I get in?’ I have asked such a question most of my life. Although I use a wheelchair more often these days, throughout my life, steps, stairs, slopes (upward and downward) have been difficult. So it is a dominant question in my lived experience.

(Like many) I thought that with better awareness around access issues and legislation via the Equality Act [2] and, of no lesser importance, members of diverse communities themselves often leading the way, my question would come to mind less often. Disappointingly, I still need to ask the question and, as a PPI contributor, I find myself asking more questions around inclusion and diversity.

Accessibility is wider than ‘getting in’ through the front door of a building and being able to move around without stress. It also applies to where buildings are situated. A lovely, ‘accessible’ building without its own car park or with car parks ‘less than 10 min’ walk away is, for some, a ‘no‐go area’. Once inside a building, it is not uncommon for doors into break‐out rooms and other internal rooms to not be automated. The accessibility of the front entrance, so full of promise, ends just inside. Quite often reception desks are too high for a seated position. Is it too difficult to design a desk that can accommodate the needs of us who operate in the world from less than average height? I and so do you know the technology exists to automate doors, so why is it not applied? In my view, access is about enabling my freedom to enter and leave as I choose.

As a PPI contributor, both nationally and locally, I have experienced many instances where my access was hindered. For example, when I attended a training event where I had to park my car on top of a hill and needed help with my wheelchair to get down the hill to enter the venue and back up the hill at the end of the day. Or when I could not push myself up a slope designed for wheelchair users. It was very steep, and the automatic door kept closing when I was halfway up. Apparently, I was not quick enough either. On that occasion, with no one available to help me, I tried to push myself round to the other entrance. This was not so easy, and I got stuck, in my chair, in a very cold wind for about 20 min until a passerby rescued me. Needless to say, I was late. In another venue, an old country house, claiming to be accessible, I could not open the big beautiful oak door to the accessible loo! Door is not automatic! I had to ask someone passing by to help. The way out was a little easier, pushed with brute force. A colleague said to me ‘you know people don't mind helping’. I do realise that, and people are, in the main, helpful and kind but I mind. I would prefer not having to ask ‘can I get in’ when invited to contribute to the research community. When access is thoroughly thought through, ‘getting in’ and ‘being there’ begins to become inclusive.

These barriers not only impact individual PPI activities but also have long‐term effects, making it more difficult for disabled people to live independently—a concern echoed by many researchers. Disabled people remain one of the most underrepresented and underserved groups in medical research, which drives health inequalities [3]. This is evident, for example, in the lack of research on essential preventive diagnostics, such as breast cancer screening for disabled people. As a result, people continue to be denied access to life‐saving health screening, such as mammography, due to the lack of investment in mammography machines designed to accommodate diverse body types [4].

How do we start to resolve some (hopefully, all) of these issues? As a PPI organiser, ask participants whether they have any special needs, listen to what is said and ask for guidance on how you can help. Check any assumptions you may have about disabled people. And remember that, to achieve good accessibility and inclusion it is sometimes necessary to do things differently. Access is not rocket science but, to me, it is beginning to feel like those involved in doing science cannot get their heads round it. Let's begin to change it together.

### Agata: No Adjustments Are Unreasonable

As a researcher responsible for planning and organising PPI, I rely on guidance on bridging the gap between research aims and questions and the public, who may be affected by the research topic or its findings. I see fighting ableism as fundamental to working in an inclusive and equitable way. The Equality Act 2010 stipulates that, where an individual meets the definition of a disabled person, there is a requirement to make reasonable adjustments to any elements that place a disabled person at a substantial disadvantage compared to nondisabled people [2]. The Act permits consideration of factors such as the cost and practicability of making an adjustment, as well as available resources, to determine what is reasonable [2].

The concept of reasonable adjustments has been applied to the involvement of disabled people in research, as seen in the recent NIHR Disability Framework, which recognises that adjustments should be co‐developed with those who require them [1]. However, the term ‘reasonable adjustments’ has been critiqued. For example, Guoxin Ma argues that the policy places an undue burden on disabled people by requiring them to be aware of their rights and how to exercise them, understand what constitutes ‘reasonable’ adjustments, and have the confidence to engage in negotiations [5]. Moreover, Ma suggests that the concept is ambiguous and often interpreted through a nondisabled lens. In other words, it relies on the judgement of an ableist society and is therefore likely to perpetuate exclusion and marginalisation [5].

The situation for PPI contributors may exacerbate these issues. Without the protection of a formal contract, disabled PPIE contributors may feel disempowered to request adjustments. Furthermore, the financial and time constraints of research projects can limit what is deemed ‘reasonable.’ In addition, the NIHR Disability Framework notes that, while 30% of its public committee members have reported a disability, only 3% of current professional committee and panel members have identified as disabled [1]. Such a disparity may increase the risk that reasonable adjustments will be defined predominantly from a nondisabled perspective. Although PPI contributors may have the skills, knowledge, and confidence to self‐advocate, this advocacy adds to the emotional labour they already provide by drawing on personal experiences to inform and advance the research.

During one of our early meetings, Sandra highlighted the importance of embedding a duty of care in PPI activities. She pointed out that while researchers are protected by workplace safety legislation, public contributors do not enjoy the same safeguards. The duty of care could address the unique vulnerabilities faced by disabled PPI contributors if embraced as a personal commitment by university staff. This means viewing public contributors holistically—understanding their abilities, preferences, needs, and history. It begins with asking how best to support them in their role, aiming to prevent harm, including emotional harm, caused by discrimination, past traumas, or feeling unheard.

This approach shifts the perspective away from interpreting needs through a nondisabled lens. It fosters a space for proactive discussions, eliminating the burden on disabled contributors to constantly advocate for their rights. Over time, placing the duty of care at the heart of our work can help us think about accessibility as an essential part of everything we do. As Sandra aptly says, whenever we plan something, we must ask ourselves: if we do it this way, who are we excluding?

## Author Contributions

**Sandra Paget:** conceptualisation, writing–original draft, writing–review and editing. **Agata Pacho:** writing–review and editing, writing–original draft.

## Ethics Statement

The authors have nothing to report.

## Conflicts of Interest

The authors declare no conflicts of interest.

## Data Availability

The authors have nothing to report.
